# On Certain Conditions of Dead Teeth

**Published:** 1883-10

**Authors:** C. S. Tomes


ARTICLE IX.

### ON CERTAIN CONDITIONS OF DEAD TEETH.

(Continued.)

BY C. S. TOMES.

(Read before the Odontological Society of Great Britain.)

To recapitulate. I believe that in the treatment of dead teeth we have to combat not only the troubles which arise from the escape of septic matter from the apex of the root, which everybody recognizes, and in a way understands, but also disease of the alveola-dental periosteum, induced in some other way, which is less frequent but more intractable. If the surmise that the alveo-dental periosteum does become diseased in other ways than by the escape of septic material from the apical root canal be true, it is difficult to see by what channel evil influence may approach it other than by the protoplasm of the cementum. This again would hardly be reached except through the medium of the contents of the dentinal tubes in the dentine of the root. Such an evil influence may, perhaps, be septic, *i. e.*, the putrefaction of the soft parts of the dentine may poison those of the cementum, and this would seem to be the most likely thing; or the destructive influence of such agent as arsenic may be propagated through the same tissues and bring about similar results in the cementum. In either case we shall have to look to the dentine of the root as the proximate cause of the mischief.

In the absence of adequate positive knowledge, conjecture may sometimes play a useful part. This must be my apology for my imperfect and inconclusive communication.

I have sometimes employed shellac for filling roots which are so fine as to render it very difficult or impossible to fill them with the materials ordinarily in use.

If it is drawn out into fine threads, it is flexible and elastic and will pass anywhere where the very finest hair bristle will pass; at the same time it is brittle enough to break off readily without withdrawal, and so soon as no more can be introduced by the side of that already in, a drop of spirit running up by capillary attraction fixes it and converts it into a very perfect filling even in very small and curved roots. It possesses this one advantage over all other root-filling materials with which I am practically acquainted; it can be introduced even into the finest root and a perfect filling made without any danger of a piston being formed to force out septic matter from the end of the root.

A little patience and hot instruments will get it out again from a large root, and I have had to thus remove it, but it would be all but impossible to get it out of a very narrow canal, and hence its use is limited to those cases in which one feels tolerably sure of not having irritation follow the filling. This same objection, however, applies to all perfect fillings in minute roots. I leave wool, of course, out of the question, as it is simply impossible to get it up such roots; but with shellac roots can be perfectly filled which can not be filled with any other material with which I am acquainted, not excepting gold wire, as the threads of shellac are easily made of a degree of fineness not ready attainable with a very soft gold wire.—*Dental Register.*

